# Immunosensing Platforms for Detection of Metabolic Biomarkers in Oral Fluids

**DOI:** 10.3390/bios15120794

**Published:** 2025-12-02

**Authors:** Nadezhda S. Komova, Kseniya V. Serebrennikova, Anatoly V. Zherdev, Boris B. Dzantiev

**Affiliations:** A.N. Bach Institute of Biochemistry, Research Center of Biotechnology of the Russian Academy of Sciences, Leninsky prospect 33, 119071 Moscow, Russia; nad4883@yandex.ru (N.S.K.); ksenijasereb@mail.ru (K.V.S.); zherdev@inbi.ras.ru (A.V.Z.)

**Keywords:** immunosensors, antigen–antibody, electrochemical immunosensors, optical immunosensors, membrane-based immunosensors, metabolic biomarkers, point-of-care diagnostics, oral fluid

## Abstract

Widespread and simple detection of diseases and disfunctions in the body is crucial for reliable and prompt diagnostics, efficient use of healthcare resources, and improved quality of life. The presence of a large number of metabolic products in saliva, the relationship between their levels in saliva and blood, the diagnostic value of many of these compounds, and the advantages of noninvasive sampling drive interest in oral fluid as a biomatrix. This review summarizes established oral fluid biomarkers, as well as potential salivary indicators for remote health monitoring and noninvasive point-of-care diagnostics. Recent advances in the search for new solutions for sensitive and high-throughput immunodetection of biomarkers in oral fluid are discussed, along with strategies for overcoming the analytical and technical challenges associated with the salivary matrix testing. Another focus of the current review is optical and electrochemical immunosensors with an emphasis on lateral flow immunoassays for point-of-care testing due to their speed, simplicity and cost-effectiveness. Finally, future directions are discussed that may enable non-invasive monitoring of endocrine, infectious, immune, neurodegenerative diseases and other human conditions using immunoassay platforms, paving the way for personalized and accessible healthcare.

## 1. Introduction

A priority for public health systems worldwide is to provide strategies for assessing the prerequisites for the development of pathologies, as well as the early diagnosis of diseases and abnormalities [1]. Achieving this goal requires identifying and assessing the content of specific compounds in biomaterial that serve as biomarkers of adverse processes in the body. Physicians have access to a wide range of laboratory methods for examining the relevant organs and tissues, which are widely used in clinical practice [2]. These primarily include instrumental laboratory methods such as chromatographic, electrophoretic, and others. Although these methods are characterized by high sensitivity and selectivity, they have serious limitations, necessitating methodological retooling of modern medical diagnostics. Firstly, their performance needs involvement of skilled professionals, expensive equipment and enormous human and material resources. Various biological fluids are collected to carry out these studies, but blood remains the most commonly used material. However, despite regular screening to detect diseases at early stages of development, some remain undetected until obvious symptoms appear. This trend can be seen in the high prevalence of diseases affecting large populations, including diabetes, cardiovascular diseases, and various malignancies [3]. To address these issues, simple and rapid mass testing is developing, which is becoming indispensable for monitoring chronic and infectious diseases. Moreover, application of simple and rapid testing methods allows for the detection of diseases on a large scale with minimal resources, as well as the control of infectious disease outbreaks, preventing further spread of infection and promoting the efficient use of healthcare system resources.

**Figure 1:**
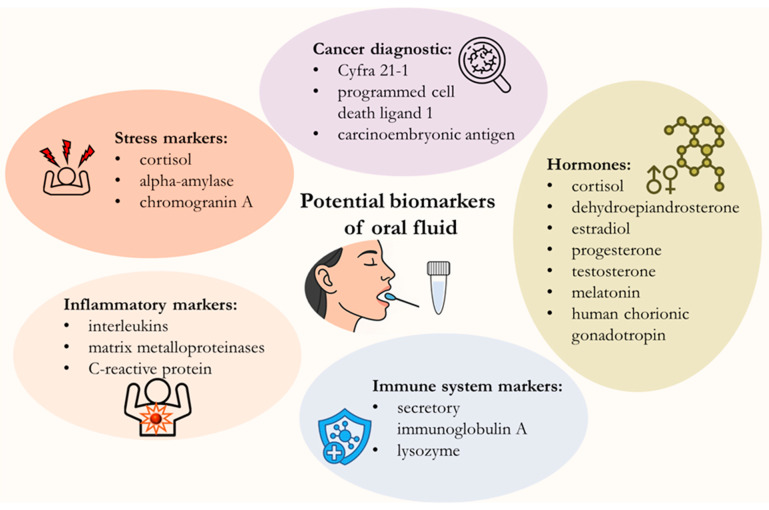
Key examples of potential biomarkers detected in oral fluid.

In this regard, non-invasive diagnostics is gaining increasing interest among mass testing methods as a practical alternative to traditional methods [4]. These methods do not require needles, surgery, or other invasive procedures, making the sample collection process more comfortable for patients and minimizing the risk of injury and infection, making them particularly valuable for long-term monitoring. The priority sample types for this purpose are saliva [5], urine [6], sweat [7] and exhaled breath [8]. Among these biomaterials, oral fluid (saliva) is the most promising sample due to the simplicity and high repeatability of the collection process [9,10,11]. In addition, saliva as a diagnostic medium is highly stable due to the use of specialized collection devices that retain its properties at room temperature for a long time [12,13]. Oral fluid is a complex mixture of various substances that perform a variety of biological functions [14]. Substances contained in oral fluid can be divided into two groups: exogenous and endogenous. Substances of endogenous origin include any biomarkers, antibodies, and metabolites produced by the body and filtered through saliva. Exogenous substances include pathogens and their fragments, allergens, drugs, potent toxic substances, pesticides, and others that enter the oral fluid from the outside. The main components of oral fluid are mucins, which are responsible for lubricating and viscoelastic properties, as well as enzymes such as amylases, proteases, and lipases, which facilitate initial digestion and play an important role in the breakdown of food and antimicrobial protection [15,16]. In addition, oral fluid contains immunoglobulin A, produced in response to microbial invasion, and other proteins (albumin, growth factors, and cytokines) responsible for protection against local and systemic diseases [17,18]. Inorganic components of oral fluid, including sodium, potassium, calcium, phosphate, and bicarbonate ions, provide pH regulation and buffering capacity of oral fluid [19]. However, the most interesting components of oral fluid as a diagnostic environment are biomarkers of endogenous origin, which reflect the systemic metabolic and hormonal states of the body. Figure 1 schematically shows the diversity of biomarkers identified in oral fluid, which confirms the potential of this biological fluid as a diagnostic environment for the assessment of pathologies and early diagnosis of diseases.

The particular value of saliva as a biomaterial for diagnostic studies is due to the abundant vascularization of the salivary glands, which ensures the rapid transfer of biomarkers from other parts of the body via the bloodstream [20]. The primary mechanism by which biomarkers enter oral fluid is passive diffusion or ultrafiltration through the capillaries surrounding the salivary glands [21]. Low-molecular-weight and lipophilic compounds (steroid hormones, drugs, and metabolites) penetrate into saliva through the membranes of acinar and ductal cells. Compounds with a higher molecular weight, such as antibodies and cytokines, can penetrate into oral fluid through the gingival crevicular fluid, which is formed from serum exudate at the gingival margin. Recent studies have shown that the secretion of specific proteins, peptides, and nucleic acids into oral fluid occurs through extracellular vesicles and exosomes secreted by epithelial and immune cells.

The concentration and composition of oral fluid biomarkers vary depending on physiological and environmental factors. Key physiological factors include salivary flow rate, circadian rhythms, hydration level, autonomic nervous system activity, as well as diet, medications, and dietary supplements [22,23]. Furthermore, it has been established that systemic diseases (endocrine, immune, and others), as well as inflammatory processes in the oral cavity, can affect the levels of salivary biomarkers. Environmental factors that influence the composition of oral fluid include stimulation of salivation and sample processing [23]. Thus, understanding the mechanisms of biomarker transfer into oral fluid and considering the factors leading to variability in its composition contribute to the reliability of oral fluid diagnostics.

**Figure 2:**
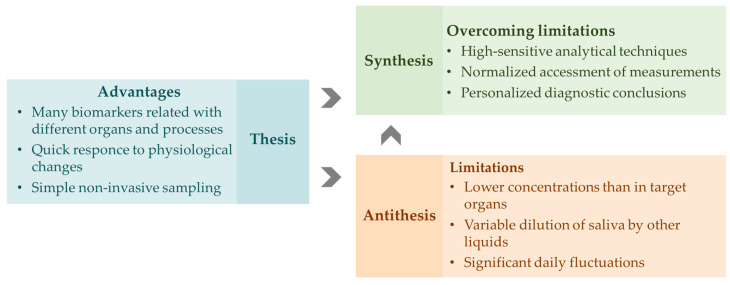
Estimation of oral fluid as diagnostic medium for biomarkers’ monitoring.

In summary, oral fluid is a promising diagnostic medium for detecting substances of both exogenous and endogenous origin. The demand for analytical developments for monitoring biomarkers in saliva has been reflected in recent reviews [11,24,25,26]. Figure 2 summarizes the advantages of oral fluid as a diagnostic medium, as well as the challenges that need to be addressed to implement immunosensing platforms in clinical practice. Therefore, the aim of this review is to summarize potential biomarkers associated with various pathologies and diseases, as well as methods for their determination based on antigen–antibody interactions. In addition, approaches to on-site oral fluid testing are discussed, including various analysis formats (membrane, microfluidic analysis) and detection methods (colorimetric, fluorometric, electrochemical).

## 2. Potential Biomarkers in Saliva

A wide range of analytes (Table 1), including hormones, cytokines, enzymes, metabolites, and immunological markers, have been identified in oral fluid, providing supportive data for the diagnosis and assessment of a person’s hormonal-metabolic, immune, or oncological status. Therefore, testing these compounds in oral fluid is an important task, as evidenced by changes in biomarker concentrations depending on pathologies and the patient’s condition. One of the most well-known salivary biomarkers is cortisol, whose levels fluctuate daily. Samples collected both in the morning and late evening have diagnostic value and allow for the assessment of stress conditions as well as adrenal function. Morning salivary cortisol levels are typically higher than late-night cortisol concentrations [27]. Decreased morning cortisol concentrations indicate adrenal disease [28,29], while elevated late-night cortisol levels are observed in patients with Cushing’s syndrome [30]. Several studies have shown that salivary cortisol levels correlate well with free cortisol in serum. Another important biomarker that complements the diagnosis of stress-related conditions and adrenal deficiency is the steroid hormone dehydroepiandrosterone sulfate (DHEA-S) [31,32,33]. In addition to assessing stress conditions, DHEA-S analysis is also used in the comprehensive diagnosis of congenital adrenal hyperplasia, adrenal tumors, and adrenal insufficiency. In addition, the cortisol/DHEA-S ratio is used in clinical practice as an indicator of the relative activity of both hormones and a marker of persistent psychiatric disorders and depressive states [34]. In this case, non-invasive oral fluid analysis has significant advantages over serum, as it allows for multiple samplings throughout the day and allows for monitoring marker fluctuations at different times of the day.

Interleukins refer to a specific type of cytokines and are present in the oral fluid of healthy individuals at low or undetectable concentrations. Elevated levels of interleukins (IL-6, IL-8, IL-10, IL-17, IL-21, and IL-22) are important in inflammatory and neoplastic processes, including periodontitis [35,36] and oral cancer [37]. Analysis of the interleukin profile in oral fluid reveals the local inflammatory status in the oral cavity but also allows for the detection of systemic immune responses [38]. This highlights the potential of salivary interleukin analysis for monitoring disease progression and response to therapy [39]. An alternative biomarker reflecting the state of the immune system in autoimmune diseases and other pathologies is secretory immunoglobulin A (sIgA). Compared to monomeric IgA found in serum, sIgA has a more complex structure—a dimeric or polymeric IgA antibody with a joining chain and a secretory component [40]. The protective function of sIgA is to neutralize pathogens and toxins on the mucosal surface, while the function of serum IgA may include the activation of immune cells via its receptor. Diagnostic applications of sIgA in oral fluid include assessing local immunity [41], diagnosing infectious diseases by testing specific sIgA [42], and identifying autoimmune processes such as systemic lupus erythematosus [43,44].

Glucose and the glucose homeostasis biomarker alpha-amylase are objects of increased interest among clinicians for non-invasive analysis of their levels in oral fluid in patients with diabetes. Salivary glucose and alpha-amylase levels have been shown to be elevated in patients with diabetes, with glucose levels correlating with blood hyperglycemia [45,46]. Simultaneous testing of these biomarkers offers an alternative method for noninvasive monitoring of the glycemic profile and stress-related state in patients with diabetes [47,48]. Furthermore, salivary alpha-amylase serves not only as an indicator of glycemic status but also as a stress biomarker due to its sympathetic regulation [49,50].

Steroid hormones represent another promising group of biomarkers for noninvasive diagnostics. Saliva testosterone levels correlate with serum free testosterone levels and are used to diagnose androgen status in both men and women, as well as children [51,52]. Moreover, salivary testosterone allows for direct measurement of bioavailable hormone, bypassing the protein binding issues associated with serum assays [52,53]. Similarly, estradiol and progesterone in oral fluid vary throughout the menstrual cycle and, in the case of estradiol, correlate well with its serum level [54]. Some studies note the usefulness of salivary estradiol measurements for monitoring its changes and preventing the risks of ovarian hyperstimulation during assisted reproductive treatment [33,55]. Additionally, sex hormone testing in oral fluid may be useful in behavioral medicine for non-invasive monitoring of hormonal changes affecting women’s physical and psychological health [33,56]. Additionally, the study [57] found that low levels of progesterone in saliva at 28 weeks of pregnancy may indicate a risk of preterm birth. Therefore, sex hormone testing in oral fluid is essential throughout pregnancy.

As Table 1 shows, in addition to the listed biomarkers, oral fluid also contains metabolites such as lactate and pyruvate, which reflect metabolic response, as well as micronutrients (vitamin D). To sum up, although oral fluid analysis allows for convenient and noninvasive monitoring of a variety of biomarkers, further research is needed to establish their clinical utility [58,59].

**Table 1:** Key biomarkers of interest in oral fluid and their diagnostic values.

Biomarker	Reference Range in Saliva	Diagnostic Value (Clinical Indications)	Ref.
alpha-amylase	Varies from 2 to 900 U/mL	non-invasive monitoring of glycemic status; clinical management of diabetic patients; biomarker of stress	[47,49,60]
Calprotectin	low or undetectable	plays a role in subclinical oral manifestations of inflammatory bowel disease; early detection of intestinal inflammation	[61]
Chromogranin A	levels fluctuate based on time of day, physiological stress, and individual health conditions	Increases with mental and physical activity or acute stress	[62,63]
Cortisol	Morning salivary cortisol: 3.0–21.0 nmol/L (median 8.4 nmol/L) Late-night salivary cortisol: 0.5–2.5 nmol/L (median 0.8 nmol/L)	elevated late-night salivary cortisol levels indicate potential Cushing’s syndrome; lowered morning salivary cortisol levels indicate adrenal insufficiency	[28,30,64]
Cortisone	vary significantly by time of day; detectable in the range of 1.2 to 100 ng/mL	elevated late-night salivary cortisol levels indicate potential Cushing’s syndrome; useful surrogate for serum free cortisol;	[30,65]
Cotinine	significant exposure: >1 ng/mL; minor exposure: <1 ng/mL	biomarker of tobacco smoke exposure	[66,67]
C-reactive protein	low, often below 1 ng/mL, or undetectable level	cardiovascular disease; autoimmune disorders; inflammatory diseases	[68,69]
Dehydroepiandrosterone sulfate	0.6–7.4 ng/mL for females and 0.6–10.1 ng/mL for males	increased levels indicate depression and anxiety; assisted marker of adrenal diseases	[31,32]
Estradiol	varies throughout the menstrual cycle and hormonal status, sub-pg/mL range	assisted reproduction monitoring; warning marker of ovarian hyper stimulation syndrome; marker of physical and psychological health	[55,56,70]
Free testosterone	vary significantly by age and sex, ~50–150 pg/mL for men ~10–50 pg/mL for women	androgen deficiency	[51,52,71,72]
Glucose	In non-diabetic group ~0.5–1 mg/mL	non-invasive monitoring of glycemic status; clinical management of diabetic patients	[47,73]
Hepcidin	Positive iron deficiency anemia: above 5.5 ng/mL; Negative iron deficiency anemia: 1.56 ng/mL < Hepcidin < 5.5 ng/mL	reflects iron regulation control of iron absorption assessment of iron bioavailability in circulation	[74,75]
Insulin	Fasting cut-off value ∼16 pmol/L	indicates consumption of glucose or mixed high-carbohydrate foods	[76]
Interleukins	low or undetectable	periodontitis oral cancer	[37,77]
Lactate	0.1–2.5 mM	monitoring of physiological conditions health monitoring	[78,79]
Lysozyme	1–57 μg/mL	indicates metabolic and microbial characteristics; stress various diseases (inflammatory bowel disease, type 2 diabetes, periodontal diseases, Sjögren’s syndrome, etc.)	[80,81]
Melatonin	vary significantly by time of day and individual factors, daytime levels range from 1 to 10 pg/mL	potential association with inflammatory markers; potential biomarker of early-stage oral squamous cell carcinoma	[82,83,84]
Progesterone	varies throughout the menstrual cycle, sub-ng/mL range	monitoring ovulation; predicting preterm birth	[54,57,85]
Pyruvic acid	-	assisted marker in cancer screening	[86,87]
Secretory immunoglobulin A	100–900 µg/mL	intestinal immune induction; potential tool in the clinical management of systemic lupus erythematosus patients; assessment of immune status	[41,43,88,89]
Tumor Necrosis Factor Alpha	low or undetectable, vary based on age, gender, and health status	oral squamous cell carcinoma; oral submucosal fibrosis; various inflammatory conditions	[90,91]
Vitamin D	varies in the range from units to hundreds of pg/mL	potential marker of oral immunity	[92]

## 3. Current Techniques for Biomarker Detection in Oral Fluid

The use of oral fluid in clinical diagnostics has become possible thanks to the development of analytical methods that allow for the reliable, sensitive and specific detection of high-molecular and low-molecular compounds. Liquid chromatography-tandem mass spectrometry (LC-MS/MS) has become a preferred technique for analyzing metabolites and protein markers in oral fluid [33]. This technique allows for the accurate identification and quantification of peptides, proteins, and small molecules. Moreover, MS allows for the simultaneous detection of multiple biomarkers of interest, which is a significant advantage over immunoassay methods. Furthermore, cross-reactivity is eliminated when analyzing biomarkers using MS. Proteomic and metabolomic analyses using LC-MS/MS has identified specific salivary biomarkers associated with cancer [93,94,95], Sjögren’s syndrome [96,97], and neurodegenerative diseases such as Alzheimer’s disease [98]. However, this technique requires sophisticated equipment and technical expertise to perform the analysis, making it more suitable for specialized laboratories.

Immunoassay techniques were developed to ensure the sensitive and specific detection of biomarkers in oral fluid. Radioimmunoassay, based on radioactive labels, was popular in the 1990s and was widely used to determine steroid hormones [99]. Further improvements in radioimmunoassay led to the development of enzyme-linked immunosorbent assays (ELISA), which allow for the analysis of small sample volumes and are easily implemented in a laboratory setting. ELISA is widely used to quantify salivary biomarkers such as interleukins, tumor necrosis factor alpha (TNF-α), cortisol, and C-reactive protein (CRP) [68,90,100,101,102]. Its relatively low cost and commercial availability make it suitable for routine laboratory use. However, the disadvantages of this technique include occasional cross-reactivity and the need for high-quality antibodies [103,104].

Due to the limitations of laboratory techniques and the burden on modern healthcare systems, the field of biosensor technologies is rapidly developing, offering unique opportunities for noninvasive and rapid on-site analysis of oral fluid biomarkers [105,106]. While ELISA and LC-MS/MS remain the gold standards for biomarker quantification, the introduction of biosensors expands the choice of analytical tools for biomarker testing in oral fluid, improves sensitivity and multiplexing capacity, and enables the application of diagnostic and monitoring methods in point-of-care settings. Electrochemical biosensors operate by detecting changes in electrical properties, while optical biosensors identify biomolecular interactions using fluorescence, absorption, or surface plasmon resonance [105,107].

As noted above, the most common and therefore commercially available technique for detecting biomarkers in oral fluid is ELISA. This technique is based on the specific interaction between an antigen and antibody, using an enzymatic label to detect the resulting complexes. The ability to analyze small sample volumes and diverse biological fluids makes ELISA a suitable tool for oral fluid diagnostics. This is confirmed by the wide variety of commercially available ELISA kits for oral fluid biomarker analysis from manufacturers such as BioCheck, Novus Biologicals, Elabskienze, Abcam, Salimetrics, IBL International, and others. Commercial ELISA kits are designed to detect hormones (cortisol, testosterone, estradiol, progesterone, DHEA-S, hepcidin, melatonin), enzymes (matrix metalloproteinase-8, amylase), inflammatory biomarkers (CRP, interleukins), and local immune biomarkers (sIgA). For example, Salimetrics specializes exclusively in saliva and offers practical solutions for oral fluid analysis, including proprietary saliva collection devices and buffer systems that minimize matrix effects and ensure testing sensitivity.

Commercial membrane-based immunoassays for oral fluid diagnostics and monitoring are a rapidly growing field, combining low-cost diagnostics with accessible and noninvasive sample collection. Membrane-based test systems available on the market are implemented in a lateral flow immunoassay format (LFIA), where all components are pre-impregnated onto the membrane, requiring only dipping the strip into the sample. Lateral flow tests for cortisol (for stress monitoring), CRP (for inflammation diagnostics), and SARS-CoV-2 antibodies, developed and manufactured by Abingdon Health and OraSure Technologies, are currently available to consumers. Furthermore, Abingdon Health has launched a new product, Salistick, the first salivary pregnancy test promising highly accurate detection of the pregnancy hormone β-hCG in saliva.

In summary, the diversity of salivary biomarkers, the ability to non-invasively collect saliva, and methodological innovations make salivary diagnostics a rapidly evolving area. The urgency of commercializing salivary diagnostic test systems underscores the need for non-invasive, simple, and easy-to-use tools for early detection and monitoring, suitable for use at home or in resource-limited settings.

## 4. Sample Collection and Pretreatment

**Figure 3:**
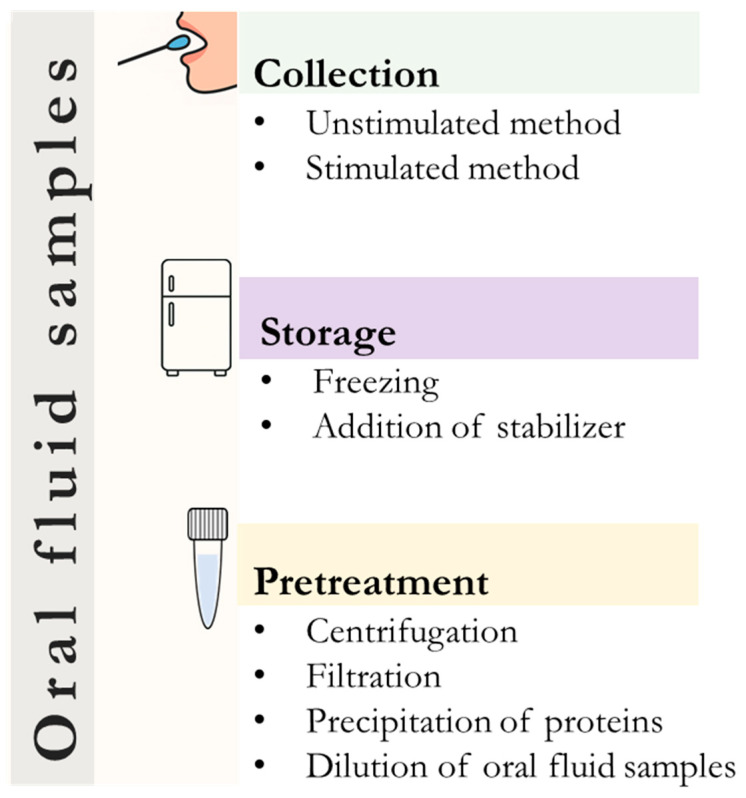
Oral fluid processing scheme, including the stages of sample collection, storage and pre-processing.

When using immunosensors for saliva diagnostics, it is important to consider the characteristics of oral fluid, as its complex composition and high susceptibility to environmental influences require careful selection of the collection method and pre-treatment (Figure 3).

Oral fluid collection methods can be divided into unstimulated and stimulated, with the choice of collection method depending on the type of assay and the component being analyzed. Collection of natural (unstimulated) oral fluid generally does not alter the sample composition and minimizes dilution of target analytes, making this method preferred in clinical and laboratory studies. This collection method is primarily used to analyze key biomarkers such as cortisol or immunoglobulin, which are particularly sensitive to stress and environmental influences. In contrast, stimulated sample collection of oral fluid relies on physical or chemical stimulation of the oral cavity, which reduces sample collection time. However, this collection method can change the pH, ionic strength, and component composition, and requires trained personnel and specialized equipment. Commercial oral fluid collection devices are increasingly being used to standardize sampling methods. For example, Lashley cups, cotton swabs, or syringes are often used to collect unstimulated saliva [108,109]. For collecting stimulated saliva, polyethylene tubes or sialographic cannulas are commonly used, as are saliva collection tubes or citric acid-soaked swabs to stimulate the glands [110]. Additionally, in the absence of the possibility to conduct the analysis immediately after sample collection, aliquots of oral fluid are frozen at −20 °C or −80 °C using stabilizers to prevent degradation [111].

The next important step after collecting oral fluid is pretreatment, which ensures the accuracy and reliability of immunosensors. The most common methods for pretreatment of oral fluid are centrifugation and membrane filtration, which remove extraneous components such as cells, large mucin deposits, and other interfering components. Furthermore, the use of immunosensors for the analysis of biomarkers in oral fluid usually requires the addition of chemical reagents to buffer the sample to the required pH and ionic strength. Additionally, the introduction of organic solvents is necessary to dissolve or precipitate large molecules such as proteins and lipids [112,113]. However, when using chemical pretreatment of oral fluid samples, the following factors should be considered: (1) sample dilution to prevent its concentration from exceeding the working range of the immunosensor; (2) careful selection of precipitation reagents to reduce adverse reactions with antibodies and labels; (3) centrifugation regimens and filtration methods to minimize the removal of protein complexes required for detection of the target analyte. Therefore, pretreatment protocols for oral fluid samples should be optimized based on not only the characteristics of the biomarker being analyzed but also the chosen assay.

## 5. Non-Invasive Oral Fluid Analysis Using Immunosensors

The development of immunosensors that combine high sensitivity, speed, and ease of use for noninvasive diagnostics of biomarkers in oral fluid is a current research area. This section of the review covers the latest developments in immunosensors, combining advances in optical and electrochemical immunosensors, as well as membrane immunosensors and microfluidic chips, which are among the priority diagnostic formats used in point-of-care settings. Optical and electrochemical immunosensors convert antigen–antibody interactions into optical and electrical signals with high sensitivity and compatibility. Integration of immunosensors with portable devices and miniature electrodes enables quantitative analysis, while data processing using smartphones enables the creation of new diagnostic solutions. High analytical sensitivity and specificity are achieved through the use of new nanolabels, antifouling coatings, and signal enhancement strategies in immunosensor designs.

### 5.1. Development of Optical Membrane Immunosensors

**Figure 4:**
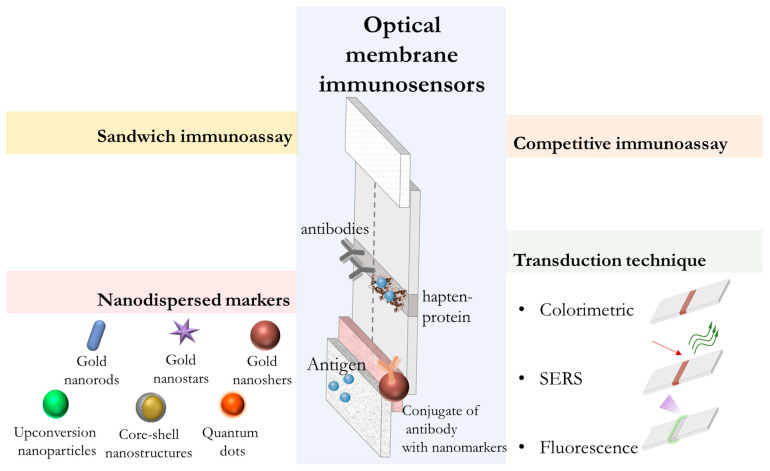
Schematic illustration of optical membrane immunosensors.

LFIA is the dominant point-of-care assay format due to its low cost and simplicity. However, performing oral fluid assays requires expanding the capabilities of traditional colorimetric LFIA. LFIA relies on capillary movement of a fluid sample along a porous membrane strip, where recognition elements (antibodies) capture the analyte and generate a detectable colorimetric/fluorescent signal. LFIA platforms include sandwich assays for larger antigens (proteins and pathogens), competitive assays for small molecules (steroid hormones), and multiplexed assays for multiple analytes (Figure 4).

The main advantages of LFIA are high assay speed, low cost, no need for expensive equipment, and the ability to integrate with digital reading systems such as smartphone cameras or portable detectors. However, in recent years, extensive research into the development of LFIA has been driven by two main goals: expanding the capabilities of non-invasive testing using saliva and overcoming the traditional limitations of sensitivity and matrix-related interference inherent in rapid test kits. Table 2 summarizes optical membrane immunosensors for the detection of biomarkers in oral fluid.

An important trend in improving the performance of LFIA for oral fluid biomarkers detection is the implementation of signal enhancement strategies beyond standard colorimetric LFIA, including the integration of plasmonic nanostructures of various natures, post-analysis enzymatic/nanozyme and chemical amplification, magnetic preconcentration, electrochemical labels and Raman scattering (SERS) tags [11,114,133]. These approaches are based either on enhancing the recorded colorimetric signal in the test line through chemical deposition of metals or the catalytic activity of nanoparticles, or on the use of additional portable instrumental detection methods (SERS, photoluminescence, digital image analysis), which can reduce detection limits. Apilux et al. applied silver enhancement to competitive LFIA of salivary cortisol by reducing silver ions on the gold nanoparticles (AuNPs) surface in the test zone. This signal enhancement resulted in a 3.6-fold increase in sensitivity and a detection limit of 0.5 ng/mL [123]. The use of bimetallic particles with nanozyme activity as nanomarkers [116,134] to improve visual colorimetric detection, as well as anisotropic Raman-labeled nanostructures to combine LFIA with SERS [129,134] have been proposed. In the study [116,134], Au@Pt was used as a marker, combining the strong localized surface plasmon resonance (LSPR) effect of AuNPs and the high peroxidase-like catalytic activity of PtNPs, resulting in a color signal orders of magnitude darker than their intrinsic color after the chromogenic reaction. It was shown that the use of Au@Pt in LFIA reduced the visual detection limit to 40 pg/mL, which is 1000 times more sensitive than before the catalytic enhancement reaction [134]. One of the recent promising achievements is based on the combination of SERS and LFIA [129]. Oh et al. synthesized size-controlled AuNP clusters and implemented them into the LFIA platform coupled with SERS detection. The achieved detection limit for cortisol in saliva was 0.014 pg/mL, which is more than 500 times higher than the sensitivity of traditional LFIA.

Conversion of immune interactions into an electrical signal is another approach to improving the performance of LFIA. Incorporating electrodes into LFIA and measuring the electrical signal using miniature potentiometers provides a lower detection limit and a wider operating range compared to traditional LFIA [130,131,135]. For example, Nandhakumar et al. [131] proposed electrochemical LFIA where detection of low concentrations of insulin were achieved by signal amplification strategy based on redox cycling. The LFIA design included a flexible screen-printed carbon electrode and a redox cycling complex consisting of a mediator (ferro/ferricyanide), a reducing agent (ammonia borane), and a catalytic label (AuNPs). Here, contributions to the electrochemical signal were provided by AuNPs-mediated nanocatalytic reduction of Fe^3+^ supported by ammonia borane on both nitrocellulose membrane of test strip and carbon working electrode, as well as redox cycling reactions at the carbon working electrode. The proposed electrochemical LFIA coupled with redox cycling amplification strategy demonstrated higher sensitivity compared to traditional colorimetric LFIA.

**Figure 5:**
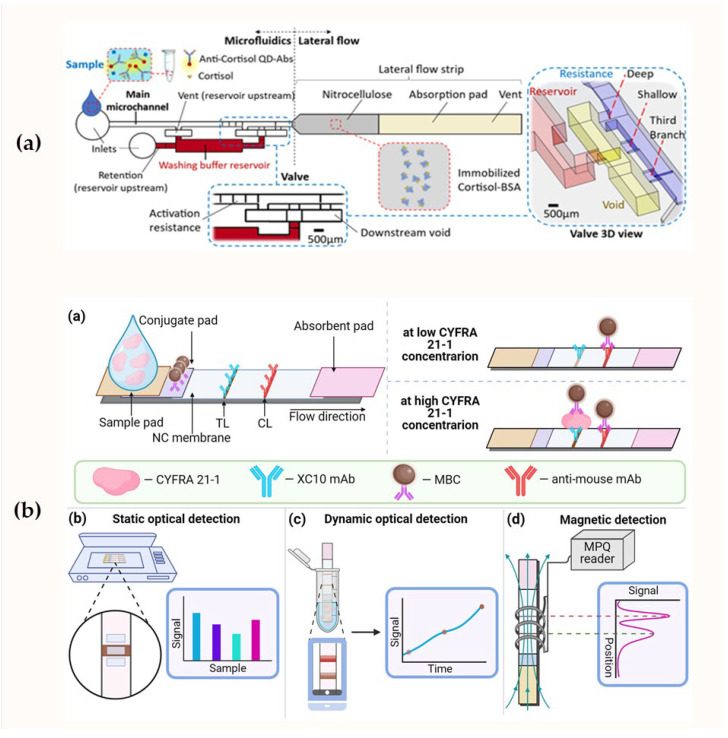
Optical membrane immunosensing platforms for oral fluid biomarkers detection: (**a**) versatile diagnostic device combined multilevel microfluidics and lateral flow assay for cortisol detection, taken from [137] with the permission of MDPI; (**b**) lateral flow assays platform using magnetic bioconjugates for CYFRA 21-1 detection, taken from [121] with the permission of MDPI, where ***a***—schematic of the test strip, ***b***—the application of various detection methods—static optical, ***c***—dynamic optical, and ***d***—electronic detection using the MPQ method.

The fluorescent reporters’ application (upconversion nanoparticles, fluorescent microspheres, platinum nanoparticles) instead of traditional colorimetric AuNPs is a popular way to improve the analytical performance of LFIA [124,125,126,127,128,136]. These labels allow for lower detection limits because fluorescence can often be measured more accurately than the intensity of colorimetric signals, and the use of additional fluorescence detection methods provides more accurate resolution of the readout images. For example, He et al. used green core–shell upconversion nanoparticles to develop a LFIA for the simultaneous detection of three biomarkers (MMP-8, IL-1β, and TNF-α), increasing the chances of timely diagnosis of periodontitis [128]. Azizian et al. proposed a novel multilayer microfluidic chip combined with a fluorescent optical membrane immunosensor platform, which provided an extended dynamic range of cortisol concentrations from 1 to 1000 ng/mL [137]. This capillary microfluidic device was fabricated using 3D printing technology and included an additional automated lateral flow system flushing step, preventing cross-contamination and reagent dilution (Figure 5a). One of the new promising markers found in oral fluid is pepsin. When released from the stomach into the oral fluid, it causes irritation and damage to the mucous membrane, leading to laryngopharyngeal reflux disease. To rapidly and noninvasively diagnose this condition, Liu et al. developed a fluorescent microsphere-based LFIA for the determination of pepsin in oral fluid with a detection limit of 1.9 ng/mL [127].

Enhancement of the analytical signal is directly related to the integration of alternative LFIA schemes for the recognition of various analytes adapted to oral fluid analysis. LFIA schemes have been proposed for the determination of low-molecular-weight hormones (e.g., cortisol) and small metabolites in oral fluid [114,117,118,122]. For example, Scarsi et al. proposed an LFIA platform for the rapid assessment of cortisol based on a color change in the test zone from blue to pink [117]. This immunosensor platform allows the identification of three main color zones that correlate with cortisol levels in oral fluid and the physiological state of the individual (normal physiological state, inflammatory response to stress, and pathological state).

To lower the detection limit of cortisol, trap-based LFIAs were developed, in which the assays have detection and deletion zones instead of test and control zones [116]. In this system, the detection zone (control zone in traditional LFIA) is the zone where the analyte concentration is measured. In contrast, the deletion zone is used to capture conjugates that do not interact with the analyte in the test zone in traditional lateral flow assays. Ultra-low concentrations of cortisol (9.9 pg/mL) were detected in oral fluid samples using the trap-based LFIA design. However, the assay time was quite long due to the time-consuming signal amplification procedure. The trap-based LFIA assay was modified to include a slow-release polyvinyl acetate tape, which maintained the ultra-low detection limit of cortisol (9.1 pg/mL) and reduced the assay time [118]. Using magnetic bioconjugates to provide higher detection sensitivity, Skirda et al. proposed a lateral flow method for detecting CYFRA 21-1 in oral fluid [121]. The combination of magnetic particle quantification and the lateral flow method eliminated signal saturation and expanded the dynamic range of detectable signals by 19 times, achieving a detection limit of 0.9 pg/mL for CYFRA 21-1 (Figure 5b).

Another area of improvement is the integration of LFIA with electronic devices or computer analysis [114,124,125,136]. The use of smartphones to capture test strip images, combined with machine learning algorithms for quantitative analysis, has significantly improved reproducibility and enabled quantitative or semi-quantitative evaluation of results. For example, a recent study used a photoluminescent film under a nitrocellulose membrane, enabling photoluminescent detection of cortisol without an external light source, followed by data processing using machine learning. The authors also confirmed good agreement between the obtained data and ELISA results [124]. A recent study [124] demonstrated the successful use of smartphone- or camera-based densitometry to capture photoluminescence images, allowing for the reliable detection of even subtle changes in color or luminescence. The implementation of such approaches improves the reproducibility and sensitivity of quantitative biomarker detection, and increases the efficiency and reliability of LFIA for point-of-care diagnostics.

**Figure 6:**
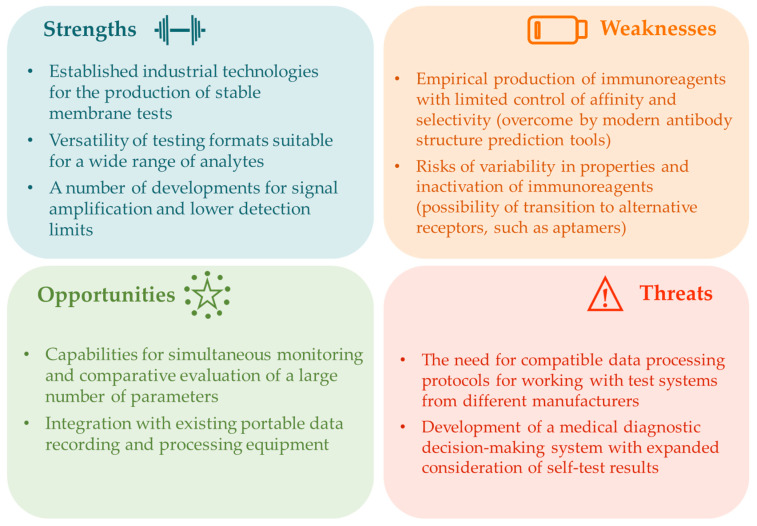
Prospects for new membrane optical immunosensors for monitoring biomarkers in oral fluid.

In summary, the above studies demonstrate that in recent years, LFIA has evolved from a high-quality diagnostic tool into a flexible platform increasingly suitable for non-invasive oral fluid testing. The described adaptations and improvements to LFIA, taken together, have opened up new opportunities for the development of sensitive and reliable LFIA methods for oral fluid analysis, suitable for rapid mass population testing and chronic disease monitoring (Figure 6).

### 5.2. Development of Electrochemical Immunosensors

**Figure 7:**
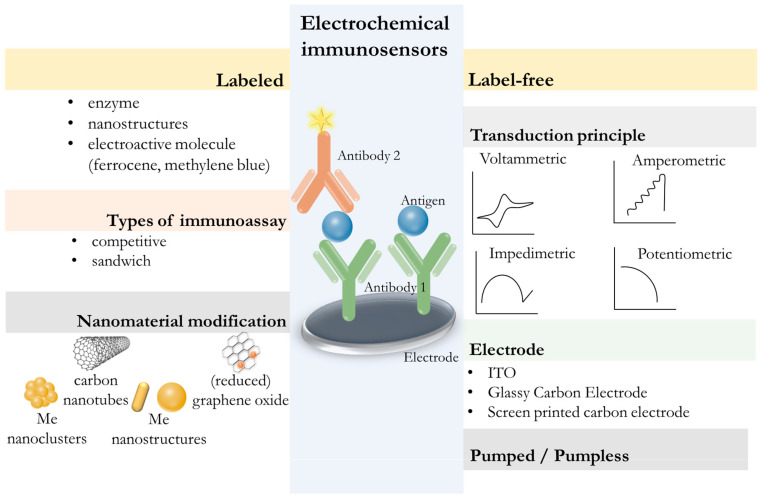
Schematic illustration of structure and operating principles of electrochemical immunosensors.

The development of electrochemical biosensors for oral fluid diagnostics has great potential due to their high sensitivity, compact design, low power consumption, and compatibility with miniature and wireless sensors [134,138]. Common methods for oral fluid analysis include amperometric and voltammetric detection of biomarkers, impedance spectroscopy/electrochemical impedance spectroscopy, and potentiometry. The choice of electrical signal conversion method often depends on the nature of the analyte and the required sensitivity of the analysis. Figure 7 shows the structure and operating principle of electrochemical immunosensors. Recent reviews [134,139,140] describe the expansion of the capabilities of electrochemical biosensors by modifying their architecture and using modern nanomaterials with diverse morphology, size, or surface charge. The use of electrodes with nanostructured coatings based on graphene/reduced graphene oxide [141,142], carbon nanotubes [143], and metal nanoparticles [143,144,145,146,147] increases the effective surface area of the working electrode and promotes faster electron transfer, improving sensitivity and signal-to-noise ratio.

The relevance and clinical significance of determining oral fluid biomarkers using electrochemical immunosensors is confirmed by the latest developments in biosensors (Table 3) for the determination of cytokines [145,148,149,150,151], matrix metalloproteases [142,152], steroid [78,144,146,153,154] and sex [155,156] hormones, and other biomarkers [157,158]. Significant improvement in analytical performance was primarily achieved through the use of nanomaterials and nanostructures in the design of electrochemical immunosensors. Graphene and reduced graphene oxide, metal and metal oxide nanoparticles (Au, Ag, ZnO), metal–organic frameworks, nanomaterials, and redox-active nanocomposites contribute to an increase in the electroactive surface area, oriented immobilization of antibodies, and acceleration of electron transfer processes, resulting in a decrease in the detection limits of target analytes (to values on the order of pg/mL or fg/mL). For example, an electroactive immunosensor based on MXene titanium carbide nanosheets decorated with silver nanoparticles (Ti_3_C_2__AgNP) and functionalized with antibodies was proposed for the determination of tumor necrosis factor-α (TNF-α) using differential pulse voltammetry [145]. Vertivel et al. synthesized a flower-structured molybdenum disulfide (MoS_2_)-decorated zinc oxide (ZnO) composite and used it in the design of an electrochemical immunosensor [159]. This composite increased the specific surface area, enhanced mass and electron transfer, and elevated the concentration of electroactive species for highly sensitive detection of interleukin-8 with detection limit of 11.6 fM. Ramos-López et al. proposed modifying a printed carbon electrode with cellulose nanocrystals, reduced graphene oxide (rGO), and p-aminobenzoic acid for oriented antibody immobilization [142]. This electrode was implemented in a sandwich immunoassay with ameprometric detection for the simultaneous determination of metalloproteinases. Modifying electrodes with quantum dots provides increased functional groups and accessibility for bioreceptor molecules, which, combined with the material’s high conductivity, ensures rapid electron transfer [160]. Modifying electrodes with nanoparticles other than quantum dots ensures more efficient electron transfer in the system. Gold nanoparticles, carbon nanotubes, titanium dioxide, and graphene are often used for this purpose. The introduction of other nanoparticles prevents charge recombination of electron carriers and, as a result, increases the sensitivity of detecting redox processes in solution. For example, Luo et al. used Ag nanoclusters as labels and CdS quantum dots modified with Au nanoparticles as photoactive receptors to create an ion-exchange platform [146]. This ion-exchange reaction between undissolved CdS quantum dots and silver ions resulted in a significant increase in photocurrent intensity, which was subsequently used to develop a competitive photoelectrochemical immunoassay for cortisol. In another study, low-toxicity CuInS_2_ quantum dots were used for sequential electrostatic adsorption, forming a CdS/melamine network on a TiO_2_-coated fluorine-doped tin oxide (FTO) electrode [78]. This composite electrode, combining the photocurrent characteristic of the TiO_2_ base with the signal enhancement of the CdS/melamine network, was used for sensitive detection of cortisol.

Along with plasmonic and semiconductor nanomaterials and nanocomposites, the use of superparamagnetic iron oxide particles also demonstrates the potential to enhance the performance of electrochemical immunosensors. Bioreceptor-functionalized magnetic micro-/nanoparticles allow for the separation of bound target analytes from unbound components and the exclusion of coexisting species from real samples in external magnetic fields, thereby increasing assay specificity. Magnetic separation at the immunoreaction stage and magnetic particle-assisted acceleration of electron transfer between the electrode and redox cites of molecules has been demonstrated in numerous developments of electrochemical sensors, including immunosensors for the determination of interleukins [148,149], cortisol [146], sex hormones [155,156] and other biomarkers [161,162].

Electrochemiluminescence-based immunoassays for the detection of ultra-low amounts of compounds are attracting increasing attention [163]. Electrochemiluminescence is based on the principle of electron transfer between electrochemically generated particles, leading to the formation of excited states and the emission of light [164]. Electrochemiluminescence immunosensors combine the sensitivity of chemiluminescence with the temporal and spatial controllability of electrochemistry, and provide virtually no background signal compared to photoluminescence. An electrochemical immunosensor based on an indium tin oxide electrode modified with a mesoporous silicon nanochannel film with dual functional domains was developed by Zhu et al. [165]. The modified electrode included an immunorecognition system on the outer surface and stable Co_3_O_4_ nanocatalysts to enhance the luminol signal within the nanochannels. The proposed sensor design combined specific recognition and catalytic systems for highly sensitive detection of IL-6 with detection limit of 0.64 fg/mL.

**Figure 8:**
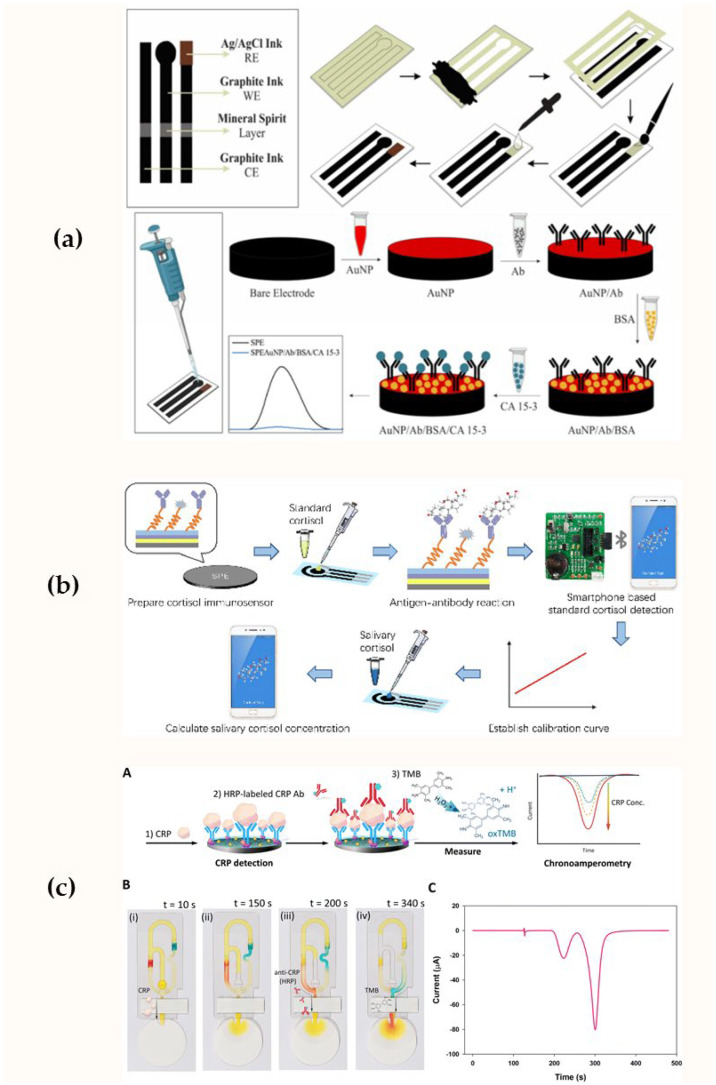
Electrochemical immunosensing platforms for oral fluid biomarkers detection: (**a**) A disposable voltammetric immunosensor using an SPE paper-based device for CA 15-3 detection, taken from [167] with the permission of MDPI; (**b**) Biosensing system based on immunosensor using gold nanoparticles/molybdenum disulfide/gold nanoparticles with the smartphone-controlled DPV detector for cortisol determination, taken from [174] with the permission of MDPI; (**c**) Capillary-driven immunoassay device for single-user step CRP detection, taken from [172], where **A**—Electrochemical detection for the CRP assay using the iceCaDI device, **B**—images of the device with 0.5% yellow food dye in running buffer, **C**—a chronoamperogram which was collected using the iceCaDI.

Unique properties of cellulose materials, such as a high surface area-to-volume ratio, high adsorption capacity, ease of chemical modification, biodegradability and biocompatibility, and fluid movement due to capillary action, have led to the emergence of a new format of paper-based electrochemical immunosensors. The functionalization of paper substrates using specialized electrode reagents, as well as controlled fluid movement in the design of a paper electrochemical immunosensor, demonstrates potential for solving problems of non-invasive diagnostics at the point-of-care settings, since this analysis format reduces costs, ensures disposability, and can be adapted for the analysis of oral fluid [153,166,167]. Tofighi et al. stabilized the synthesized Ag nanoink by printing it on a photographic paper surface using pen-on-paper technology [166]. The resulting substrate was used to develop an electrochemical immunosensor for the highly sensitive detection of the Cyfra21.1 biomarker in oral fluid. Han et al. proposed an alternative paper biosensor chip design, in which gold microelectrodes were formed on a paper substrate [153]. The electrochemical immunosensor platform was integrated with a miniature printed circuit board for wireless transmission of electrical signals and their real-time monitoring using MATLAB. The developed paper biosensor chip was used to detect cortisol in oral fluid. Oliveira et al. fabricated a screen-printed paper-based electrode using homemade conductive inks (Figure 8a) [167]. To immobilize specific antibodies, the electrode was modified with gold nanoparticles. The developed immunosensor platform was implemented for the detection of CA 15-3, using differential pulse voltammetry with potassium ferrocyanide as the redox reagent to register antigen–antibody interactions.

The concept of single-chip sensor devices for the detection and monitoring of metabolic biomarkers in a small volume of untreated salivary fluid also appears promising for point-of-care diagnostics [168]. Recent studies have demonstrated the development of chips that combine electrocatalytic and immunoassay detection principles for the simultaneous detection of vitamin C and vitamin D for physiological monitoring [169], as well as glucose and insulin for diabetes management [170]. The proposed dual sensing approach combines enzyme and antibody-based assays to simultaneously detect two markers in a drop of saliva in less than half an hour. An alternative design for multiplexed on-chip immunoassay was proposed by Moukri et al. [171]. The sensor design consisted of a lithographically fabricated three-disk gold working micro-electrodes coated with gold foam via electrochemical deposition using a hydrogen bubble template and modified with an antifouling coating comprising chitosan, GO, and BSA. The antifouling layer also ensured site-directed antibody immobilization due to its functionalization with protein A/G. The proposed sensor, based on three gold disk working electrodes, demonstrated high sensitivity for the simultaneous analysis of IgG and IL-8 in saliva. Kaewarsa et al. [172] proposed an immunoassay platform that offers portability, ease of use, and reduced analysis time through the use of a pumpless sensor design. Integration of commercial electrodes into a capillary-driven immunoassay (iceCaDI) device and improvements to the microfluidic system, such as vent holes to remove air bubbles, demonstrated the feasibility of single-step quantitative determination of CRP. However, despite the advantages of pumpless electrochemical immunoassays over pumped immunoassays such as simplicity, low cost, and portability suitable for use at the point-of-care settings, lower assay throughput due to limited flow control and lower sensitivity require further improvements of pumpless devices [173].

Miniaturization of devices are also relevant areas that are taken into account when developing electrochemical biosensors for non-invasive diagnostics of saliva. Integrating immunosensors with low-cost portable electrochemical devices and smartphone data readers makes testing more accessible. Liu et al. proposed a miniature battery-powered circuit board for operating an immunosensor based on differential pulse voltammetry (DPV) [174]. The miniaturized DPV system was connected to a smartphone, where a developed app was used to control detection, receive feedback signals, and display results in real time (Figure 8b). This smartphone-controlled DPV system, using AuNPs/MoS_2_/AuNPs-modified and antibody-functionalized screen-printed electrodes, was implemented for cortisol detection. Tamiya et al. analyzed changes in the concentration of neutralizing antibodies, IgG and secretory IgA, in saliva and serum after vaccination against viral diseases using a pocket-sized potentiostat and demonstrated the utility of the proposed solution for tracking neutralizing antibodies in response to vaccination and assessing its effectiveness [175]. A hybrid approach combining the capture of a target analyte from a sample using immunomagnetic beads and the modification of gold sensor electrodes with 3D dendritic structures made of gold nanoparticles has enhanced the performance of electrochemical detection and achieved high sensitivity for the analysis of Alzheimer’s disease biomarkers, lactoferrin, and amyloid β-protein 1−42 [162]. The detection limits of lactoferrin and amyloid β-protein covered the physiological concentrations of biomarkers in saliva and were 2 μg/mL and 0.1 pg/mL, respectively. The miniature sensor design with a detachable magnet in the working electrode region and pre-capture of the biomarker from the sample using functionalized magnetic beads demonstrates the potential of this sensor system for clinical application.

Furthermore, machine learning applied to a wealth of electrochemical data (current intensities and potentials) improves diagnostic accuracy [176]. Supervised machine learning improves the performance of electrochemical immunosensor platforms and reduces analysis time, enabling rapid interpretation of large volumes of data and the timely detection of sensor abnormalities. For example, the integration of supervised machine learning with a microfluidic impedance cytometer was used to quantify immunoglobulins G and A in oral fluid, reducing analysis time to 2 min through more intelligent automation [161].

It is important to note that validation of assays in clinical studies involving large numbers and diversity of samples, as well as reliable instrument calibration, remain critical steps before many of these technologies can be used in routine clinical practice.

**Table 3:** Electrochemical biosensors for saliva diagnostics.

Sensor Design	Working Electrode	Transduction Principle	Analyte	Detection Range	Limit of Detection	Ref.
Amperometric immunosensors						
Multiplex sandwich-type immunosensor platform	disposable carbon electrodes with four working surfaces	amperometry	IL-6, receptor activator of NF-kB ligand, protein arginine deiminase 4	IL-6: 2.0–1000 pg/mL; RANKL: 2.5–1000 pg/mL; PAD4: 0.1–1000 ng/mL; anti-PAD4: 10–1000 ng/mL	IL-6 0.09 pg/mL RANKL 0.10 pg/mL PAD4 0.09 ng/mL anti-PAD4 14.5 ng/mL	[148]
multiplexed immunoplatform with four-channel potentiostat	four working electrodes of the screen-printed carbon electrode array	amperometry	progesterone, luteinizing hormone, estradiol, prolactin	progesterone: 38.1–250.0 pg/mL; estradiol: 23.3–50,000 pg/mL; luteinizing hormone: 1.0–100.0 mIU/mL; prolactin: 1–100 ng/mL	progesterone 2.85 pg/mL estradiol 5.88 pg/mL luteinizing hormone 0.30 mIU/mL prolactin 0.32 ng/mL	[155]
miniaturized, silicon-based sensor	gold sensing electrodes, modified with AuNPs (3D dendric nanostructures)	amperometry	lactoferrin and amyloid β-protein 1-42	Lactoferrin: 2–32 μg/mL amyloid β-protein 1-42: 0.1–1 ng/mL	Lactoferrin: 2 μg/mL; amyloid β-protein 1-42: 0.1 pg/mL	[162]
dual sandwich-type immunosensor	screen-printed dual carbon electrodes modified with reduced graphene oxide and crystalline nanocellulose	amperometry	MMP-9 and MMP-13	1.0–1000 ng/mL	MMP-9 0.25 ng/mL MMP-13 0.30 ng/mL	[142]
Sandwich-type immunosensor	a screen-printed carbon electrode	chrono- amperometry	galectin-3	n/a	9.66 ng/mL	[150]
capillary-driven microfluidic immunosensor	screen-printed carbon electrode	chrono- amperometry	C-reactive protein	0.625–10.0 μg/mL	0.751 μg/mL	[172]
A dual bioelectronic sensor chip	two Au-sputtered electrodes	chrono- amperometry	vitamin C and vitamin D	vitamin C: 0–800 μM; vitamin D: 0–200 ng/mL	vitamin D: 29 ng/mL in buffer and 12 ng/mL in saliva; vitamin C: 28 μM in buffer and 84 µM in saliva	[169]
A dual-marker biosensor chip	two Au electrodes	chrono- amperometry	Insulin and glucose	Glucose: 0–20 mM; Insulin: 0–1200 pM	glucose: 0.2 mM; insulin: 41 pM	[170]
disposable immunosensing platforms using a field-portable dual potentiostat	disposable screen-printed carbon electrode	amperometry	luteinizing hormone and progesterone	luteinizing hormone: 0.3–125 mIU/mL; progesterone: 10^−2^–10^3^ ng/mL	luteinizing hormone 0.10 mIU/mL; Progesterone 1.7 pg/mL	[156]
sandwich-type immunoplatform	cerium dioxide nanoparticles/ multi-walled carbon nanotubes composite	amperometry	myeloperoxidase	1.0–100.0 ng/mL	0.40 ng/mL	[143]
Impedance immunosensors						
digital sensor chip	micro-Au electrodes	impedance	cortisol	1 pg/mL– 10 mg/mL	0.87 pg/mL	[154]
immunosensor	gold microelectrodes biofunctionalized using 4-carboxymethyl aryl diazonium	impedance	N-Terminal Natriuretic Peptide	1–20 pg/mL	0.03 pg/mL	[157]
A sensor chip with an electrical impedance analyzer	hybrid nanocomposite of graphene nanoplatelets with diblock co-polymers and Au electrodes	impedance	Prostate specific antigen	0.0001–100 ng/mL	40 fg/mL	[141]
immunosensing strip	screen-printed carbon electrode functionalized by carbon spherical shells polyethylenimine, glutaraldehyde	impedance	brain-derived neurotrophic factor	10^–20^–10^–10^ g/mL	10^–20^ g/mL	[158]
microfluidic impedance cytometer chip (with machine learning)	two gold microelectrodes on a glass substrate with a polydimethyl siloxane channel	impedance	IgG/IgA	n/a	16.67 nM for IgG/IgA ratio	[161]
Voltammetric immunosensors						
Electroactive interface-based immunosensor	silver nanoparticle-decorated titanium carbide MXene nanosheets modified screen-printed electrode	differential pulse voltammetry	TNF-α	1–180 pg/mL	0.97 pg/mL	[145]
disposable voltammetric immunosensor	screen-printed paper-based electrode modified with gold nanoparticle	voltammetry	CA 15-3	2–16 U/mL	0.56 U/mL	[167]
Electrocatalysts-based immunosensor	glassy carbon electrode modified with flower-structured MoS_2_/ZnO composite	differential pulse voltammetry	IL-8	500–4500 pg/mL	11.6 fM	[159]
Sandwich-type immunoassay	Au electrode	differential pulse voltammetry	sIgA silica nanochannel film	10–300 ng/mL	10 ng/mL	[147]
flexible three-electrode paper-based immunosensor	three-electrode system based on synthesized Ag ink with pen-on-paper technology on the surface of photographic paper	differential pulse voltammetry	Cyfra 21.1	0.0025–10 ng/mL	0.0025 ng/mL	[166]
multiplex immunosensor	gold micro-electrodes modified by gold foam	differential pulse voltammetry	IgG and IL-8	IgG: 0.01–1 pg/mL; IL-8: 0.01–1 pg/mL	IgG: 69.2 fg/mL; IL-8: 87.6 fg/mL	[171]
Other types of immunosensors						
a paper-based electrical biosensor chip integrated with a printed circuit board	polymer– graphene nanoplatelets composite on Au electrodes	conductometry	cortisol	3 pg/mL–10 μg/mL	50 pg/mL	[153]
Sandwich-type immunosensor	glassy carbon electrod modified with ZnO nanoparticles	chronopotentiometry	cortisol	10^–7^–10^2^ nM	5 × 10^–7^ nM	[144]
biphasic sandwich-type immunoassay based on tyramine-DNA cascade signal amplification	indium tin oxide	electrochemical with tyramine-DNA cascade signal amplification	IL-8	1.0–1000 pg/mL	0.28 pg/mL	[149]
duplex of biosensors for multi-resonance analysis	Fiber-Bragg grating-assisted semi-distributed interferometer-based probe	interferometry	IL-6 and IL-8	n/a	IL-6 480 aM IL-8 23.4 fM	[152]
signal-on photoelectrochemical competitive immunoassay	CdS/Au electrode	photoelectric	cortisol	0.0001–100 ng/mL	0.06 pg/mL	[146]
competition-type photoelectrochemical immunosensor	Fluorine-tin-oxide/TiO_2_/CuInS_2_ quantum dots electrode	photoelectric	cortisol	0.01–100 ng/mL	7.8 pg/mL	[78]
Enhanced electrochemiluminescence immunosensor	indium tin oxide modified with mesoporous silica nanochannel film and Co_3_O_4_ nanocatalyst	electrochemiluminescence	IL-6	1 fg/mL–10 ng/mL	0.64 fg/mL	[165]

### 5.3. Other Optical Immunosensors

Numerous immunosensors have been developed and adapted for oral fluid diagnostics using various measurement methods, including surface plasmon resonance (SPR), surface-enhanced Raman spectroscopy (SERS) and fluorescence (see Table 4).

Optical surface plasmon resonance immunosensors have attracted particular attention due to their ability to monitor molecular interactions in real time and without the use of labels. The operating principle of plasmonic sensors is based on recording the shift in the resonant wavelength in response to a change in the refractive index in metal nanostructures as a result of biomolecular interactions [177]. Implementation of plasmonic biosensors for salivary diagnostics requires increased analytical sensitivity, which can be achieved through the implementation of the latest advances in nanotechnology and surface chemistry. For example, Kumar et al. designed a double-sided polished photonics crystal fiber-based long-range surface plasmon resonance (LRSPR) sensor where a silver layer was sandwiched between two dielectric buffer layers of MgF_2_ on both sides of the photonics crystal fiber surface, forming the LRSPR mode [178]. The developed sensor demonstrated the ability to detect physiologically relevant ranges of cortisol in saliva. However, detecting small molecules (e.g., hormones) or very low analyte concentrations often presents a challenge for label-free plasmonic immunosensors.

To address this challenge, immunosensors using fluorescent or plasmonic nanoparticles, SERS nanotags, or photothermal/aggregation effects via LSPR have been proposed. Fluorescent immunosensors combine the use of highly efficient labels, such as quantum dots or fluorescent metal nanoclusters, which provide enhanced brightness and extended dynamic range [179,180,181]. For example, Ag nanoclusters as a fluorescent label and MnO_2_ nanosheets as an effective quencher were used to develop a dual-mode fluorescent immunoplatform for the ultrasensitive and noninvasive detection of programmed cell death ligand 1 (PD-L1), a biomarker in cancer immunotherapy [182]. The quenching properties of AuNPs were exploited to construct a Förster resonance energy transfer-based immunosensor, where the interaction of antibodies on the nanoparticle surface with the target analyte (CRP) resulted in the release of photoluminescence from Zn_2_GeO_4_:Mn^2+^ nanoparticles [183]. The obvious advantages of ELISA support continued interest in this method and determine the relevance of introducing amplification strategies to improve its efficiency with the ultimate goal of expanding the capabilities of the method for routine use. For example, a progesterone ELISA based on a nanozyme label (Pt/IrO_2_@SA@HRP nanoflowers), which provides enhanced catalytic activity and stability [184], demonstrated good analytical performance and good agreement with LC-MS/MS data. Replacing the microplate ELISA format with a paper one with hydrophobic barriers applied using a laser printer and modification of antibody immobilization sites with hydroxyapatite demonstrated the potential of the proposed system for noninvasive detection of alpha-fetoprotein in oral fluid [185].

SERS biosensors using antibody-functionalized SERS nanotags can extend detection limits to the fg/mL range in saliva [186,187]. Thus, Li et al. used Au@Ag-Au core–shell nanotags in the design of a silicon chip-based SERS immunosensor to detect femtogram amounts of IL-6 in saliva [188]. Moreover, anisotropic plasmonic nanostructures, such as Au nanorods or Au nanostars, as well as nanoparticle aggregation strategies, have been implemented to enhance the sensitivity of immunosensors in lateral flow assay format [129,134] and have demonstrated orders of magnitude reductions in the detection limit of oral fluid biomarkers compared to traditional colorimetric LFIA (see Section 5.1 for more details on LFIA sensitivity enhancement strategies).

Despite the high sensitivity and multiplexing capabilities of SERS and fluorescent immunosensors, their implementation necessitates the use of spectral readout devices. In contrast, colorimetric and photothermal nanoparticle-based immunosensors enable signal acquisition using portable devices or smartphones in combination with data processing software. For example, Pinto et al. developed a microfluidic immunosensor fabricated in poly(dimethylsiloxane) for detecting cortisol in saliva [189]. Combining a microfluidic design with poly(dimethylsiloxane) and a metal-oxide-semiconductor-based silicon photodiode as a photodetector demonstrated the potential for further fabrication of a miniaturized and reusable device for noninvasive cortisol monitoring. Furthermore, Lee et al. developed a biosensor based on AuNPs conjugated with antibodies that exhibited a photothermal effect when irradiated with a 532 nm laser [190]. Recording temperature changes for each CRP concentration enabled the detection of low protein concentrations in saliva.

To improve the performance of immunosensors, designs combining highly effective labels, sensitive detection methods, and amplification strategies have been proposed. For example, the use of fluorescent labels (fluorescent dyes, up-conversion nanoparticles), readout techniques (fluorescence intensity, fluorescence resonance energy transfer) and the design of biochips using photonic crystals to enhance the signal amplitude are particularly attractive for saliva diagnostics [191]. The success of fluorescent biochips with amplification approaches is confirmed by the latest developments in immunosensors for the detection of migraine biomarker [192] and carcinoembryonic antigen [193]. Liang et al. [194] developed microfluidic immunosensing device that combines vibration-enhanced incubation module, a magnetic-assisted separation module, a microfluidic chip and colorimetric readout technique for detecting multiple biomarkers of cardiovascular diseases. This biosensor demonstrated the ability to rapidly and end-to-end detect CRP, IL-6, and procalcitonin within minutes by integrating immune magnetic bead preparation, one-step immunoassay, washing, enzymatic reaction, and colorimetric signal detection. Wang et al. proposed an AuNPs@HRP@FeMOF immune scaffold that combines the antibody adsorption and enzyme protection capabilities of a metal–organic framework (MOF), the catalytic activity of HRP, and the peroxidase-like activity of FeMOF and AuNPs [193]. Synthesized in a single step, the AuNPs@HRP@FeMOF immune scaffold was successfully applied for the colorimetric detection of Cyfra 21-1, a cancer biomarker. In addition to optical signal amplification, labels in immunosensors enable multiplexing due to their different spectral characteristics. For example, Au nanorods with tunable optical properties by changing the structure geometry were used for multiplexed colorimetric analysis of oral cancer biomarkers [195]. Two Au nanorod-based bioprobes with LSPR at wavelengths of 620 and 775 nm demonstrated distinguishable binding responses to Cyfra 21-1 and CA-125 in saliva. Fluorescent beads exhibiting different excitation and emission wavelengths were selected as labels for a multiplexed single-step immunoassay [196]. Measuring the fluorescent signal in the bound-free phase allowed for the simultaneous detection of MMP-8, MMP-9, and tissue metalloproteinase inhibitor 1 in saliva.

The presented examples of hybrid approaches in immunosensor designs are aimed at improving the testing performance and allow achieving or approaching the requirements of clinical sensitivity in combination with a microfluidic format by removing interfering components from the sample and concentrating target analytes [188].

**Table 4:** Other optical immunosensors for oral fluid diagnostics.

Sensor Design	Label	Signal Generation	Analyte	Detection Range	Limit of Detection	Ref.
sensing immune platform	AuNPs@HRP@FeMOF immune scaffold	colorimetric	Cyfra 21-1	3.1–50.0 ng/mL	0.37 ng/mL	[193]
dual-mode sensing platform	silver nanoclusters with MnO_2_ nanosheets	fluorescence	PD-L1	1.98–196.78 pg/mL	0.44 pg/mL	[182]
fluorescence resonance energy transfer (FRET)-based immunoassay	Zn_2_GeO_4_:Mn^2+^ nanoparticles and Au nanoparticles	fluorescence	CRP	5–20 ng/mL	2.5 ng/mL	[183]
colorimetric sandwich immunosensor	magnetic particles	colorimetric	insulin	10 pM–1 nM	10 pM	[197]
fluorescence competition immunoassay	photonic crystal	fluorescence	calcitonin gene-related peptide	0.05–100 pg/mL	0.05 pg/mL	[192]
turn-off biosensor based on polymethylmethacrylate opal photonic crystals	photonic crystal and AuNPs	fluorescence	carcinoembryonic antigen	0.1–2.5 ng/mL	0.1 ng/mL	[193]
* SPR-based optical biosensor system	-	optical	macrophage inflammatory protein-1α	n/a	129 fM (1.0 pg/mL) in buffer 346 fM (2.7 pg/mL) in saliva	[198]
all-in-one microfluidic immunosensing device	magnetic beads	colorimetric	CRP, IL-6, procalcitonin	CRP: 1.75–28 ng/mL; IL-6 and PCT: 1.56–100 ng/mL	CRP: 0.295 ng/mL; IL-6: 0.400 ng/mL; PCT: 0.947 ng/mL	[194]
multiplex bioanalytical assay	Au nanorods	optical	Cyfra 21-1 and CA-125	Cyfra 21-1: 0.496–48.4 ng/mL; CA-125: 5–320 U/mL	Cyfra 21-1: 0.84 ng/mL CA-125: 1.6 U/mL	[195]
wash-free bead-based immunoassay	magnetic beads and fluorescent beads	fluorescence	CRP, MMP-8 and MMP-9 and TIMP-1	CRP: 20–140 mg/L; MMP-8: 0.47–30 ng/mL; MMP-9: 0.47–30 ng/mL; TIMP-1: 0.69–44 ng/mL	CRP: 2.1 ± 6.3 mg/L MMP-8: 0.24 ng/mL MMP-9: 0.38 ng/mL TIMP-1: 0.39 ng/mL	[196]
double-sided polished photonics crystal fiber-based SPR sensor	-	optical	cortisol	1.2–30 ng/mL	1.2 ng/mL	[178]
a photothermal biosensor	AuNPs coupled to a platinum detector	temperature change	CRP	0.1–100 ng/mL	0.1 ng/mL	[190]

* SPR-surface plasmon resonance.

## 6. Future Perspectives

**Figure 9:**
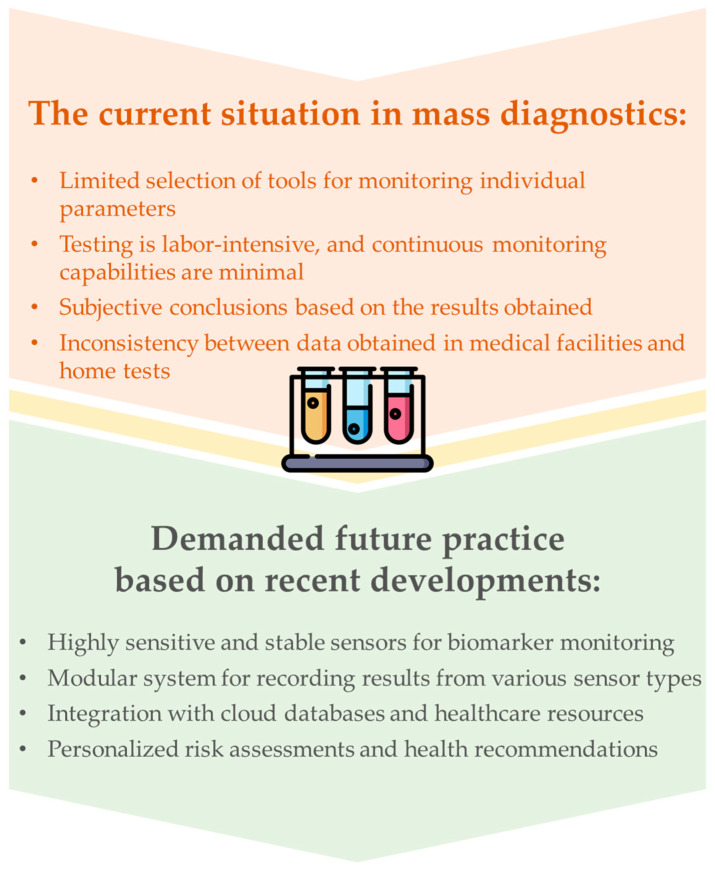
State-of-the-art and perspectives of diagnostics based on biomarkers monitoring in oral fluid.

Despite significant progress in development of immunosensing platforms for oral fluid diagnostics, a number of challenges remain associated with their implementation in clinical practice (Figure 9). Oral fluid is a readily accessible biological matrix and is the preferred medium for noninvasive diagnostics. However, the multicomponent composition and variability of saliva properties under the influence of various factors hinder achieving the analytical reliability and diagnostic accuracy necessary for clinical decision-making. The physicochemical variability of oral fluid is a key challenge and is caused by fluctuations in the composition and properties of oral fluid. Unlike serum or plasma, oral fluid is characterized by low analyte concentrations (99% of saliva is water, and the remaining 1% is dissolved organic and inorganic compounds), variable viscosity, and density, and is subject to change depending on age, nutrition, health status, oral hygiene regimen, and the presence of any pathologies [23,199]. The listed factors have a significant impact on the reproducibility and sensitivity of immunosensors, which can be prevented by controlling the preanalytical stage of the study and optimizing the analysis conditions to ensure reliable quantitative assessment of biomarkers in oral fluid [200,201,202].

In addition to physicochemical properties, saliva components such as mucins, enzymes, and electrolytes can also interfere with antigen–antibody interactions, the catalytic properties of enzyme or nanozyme labels, and optical or electrochemical signal detection [175,188]. In this case, the development of immunosensing platforms should be accompanied not only by adapting antibody affinity and immune interaction conditions [122,203] but also by implementing effective preprocessing techniques for oral fluid samples [119,204]. Oral fluid preprocessing can include centrifugation to separate solid particles from the liquid fraction, filtration to separate extracellular proteins and nucleic acids, amylase depletion, or the addition of stabilizing agents. However, it is worth noting that saliva preprocessing complicates the analysis procedure and transforms this assay format from a simple noninvasive diagnostic to a laboratory method. Furthermore, the lack of standardized devices for saliva sampling and general protocols for sample collection, storage, and processing of saliva remains a weakness in immunoassay-based oral fluid diagnostics [205,206]. Commercially available oral fluid collection devices vary in buffer composition, reconstitution volume, and sample storage periods, leading to variability in test results. Therefore, establishing universal standards for collection, storage, and analysis is crucial for integration of immunosensing platforms into diagnostic practice for the metabolic biomarkers diagnostics in oral fluid, ensuring reproducible results and clinical reliability.

Improving sensitivity and specificity is another pressing challenge in development of immunosensing platforms, as trace concentrations of biomarkers in oral fluid require the implementation of amplification approaches to lower detection limits of target analytes down to picogram or femtogram levels. Combining immunosensors with effective labels and highly sensitive detection methods, such as chemiluminescence, electrochemistry, and surface-enhanced Raman spectroscopy, has improved sensitivity [207]. However, achieving clinically relevant thresholds for specific analytes requires further refinements. Eliminating cross-reactivity and nonspecific binding to prevent false-positive or false-negative results also remains a challenge that must be addressed [208]. Furthermore, the instability of immunoreagents and nanodispersed marker-antibody conjugates with changes in temperature or pH in saliva samples adds additional complexity to the application of immunosensing platforms for oral fluid diagnostics [33].

Translating immunoassay platforms from laboratory development to real-world point-of-care diagnostic applications is the ultimate goal of research [209]. While a lot of immunosensors demonstrate high performance under standard conditions, reliability drops sharply in decentralized or point-of-care settings. Ensuring assay stability and accuracy, along with user-friendliness and cost-effectiveness, therefore requires a comprehensive approach to immunosensor engineering. Miniature read-out devices and microfluidic chips are attracting particular attention due to their ability to analyze small sample volumes, automate multiple analysis steps, and process data on-site [168,210]. However, large-scale production of such devices is hampered by low throughput and the lack of standardized quality control with biomarker levels in oral fluid acceptable for clinical diagnostics. Furthermore, there is currently a lack of research establishing diagnostic thresholds for biomarkers in oral fluid and their correlation with blood levels.

To address these challenges and limitations in adapting immunoassay platforms for oral fluid diagnostics, several promising research areas have been proposed, including the production of nanomaterials with unique physicochemical properties and the engineering of receptor molecules. For example, the use of plasmonic nanomaterials (Au and Ag nanoparticles), photonic crystals, quantum dots, and magnetic particles has demonstrated the potential to enhance specificity and reduce nonspecificity, as well as improve the sensitivity and dynamic range of analysis [191,211,212,213]. Advances in computer design, as well as molecular biology and engineering methods, enable the development of recognition reagents with enhanced affinity, specificity, and stability, such as single-chain variable fragments (scFv), nanobodies, and aptamers, which meet the criteria required for working with such a complex diagnostic medium as oral fluid [214]. In addition, the integration of immunoassay platforms with smartphone-based digital data detection and processing systems has been proposed, which could potentially facilitate the translation of immunosensors into a point-of-care testing format and their use in resource-limited settings for diagnostics and health monitoring [214].

The integration of immunosensing platforms with data processing using artificial intelligence and machine learning also demonstrates potential for identifying biomarkers in saliva and interpreting the resulting data [215]. This processing is particularly relevant for multiplex analysis. Correlating immunoassay results with the proteomic and metabolomic profile of saliva using artificial intelligence has the potential to improve diagnostic accuracy and enable personalized monitoring and disease risk assessment. Furthermore, the discovery of new oral fluid biomarkers and their relationships with blood parameters, as well as the development of new immunosensing platforms to identify new classes of biomarkers, could expand the clinical value of immunosensors for oral fluid testing.

## 7. Conclusions

This manuscript presents an overview of biomarkers detected in saliva, their correlation with various diseases and conditions in the human body, and immunoassay platforms based on optical and electrochemical readout techniques. Various strategies aimed at immunosensing platforms for diagnostics in oral fluid are discussed, including amplification approaches based on new materials and signal detection methods, sample preparation methods, and sensor designs.

In summary, despite the existing challenges, development of immunosensing platforms for use in oral fluid is a promising area of research and holds significant potential for noninvasive clinical diagnostics. Developing new amplification approaches, standardizing oral fluid collection and sample preparation protocols, and designing cost-effective and easy-to-use devices combined with automated data processing remain pressing challenges. Furthermore, validating the effectiveness of proposed immunosensing platforms in clinical practice using large samples across diverse populations is crucial for translating developments from experimental prototypes into diagnostic systems for monitoring and assessing disease risk in point-of-care settings.

## Figures and Tables

**Table 2:** Lateral flow immunoplatforms for oral fluid diagnostics.

Sensor Design	Label	Signal Generation	Analyte	Detection Range, ng/mL	LOD, ng/mL	Ref.
lateral flow immunoassay	gold nanoparticles	colorimetric	cortisol	0.01–10	3.8 × 10^−3^	[114]
paper-based microanalytical devices	gold nanoparticles	colorimetric	cortisol	n/a	n/a	[115]
trap lateral flow immunoassay based ratiometric method (with deletion and detection zones)	gold nanoparticles	enzyme-catalyzed color signal	cortisol	0.01–100	9.9 × 10^−3^	[116]
dual-color competitive lateral flow immunoassay	gold nanoparticles and gold nanostars	color transition from red to blue	cortisol	n/a	low (0–10 ng/mL)—blue signal medium (15–25 ng/mL)—more purple, high (30–50 ng/mL), clearly pink/reddish	[117]
paper-based trap lateral flow immunoassay	gold nanoparticles	colorimetric	cortisol	0.01–1000	9.1 × 10^−3^	[118]
immune-chromatographic strip	gold nanoparticles	colorimetric	pepsin	10–5000	10	[119]
Immune- chromatographic test	gold nanoparticles	colorimetric	cortisol	1–100	1	[120]
lateral flow immunoassay	magnetic particles	colorimetric	CYFRA 21-1	0.002–2.1	0.9 × 10^−3^	[121]
lateral flow immunoassay	gold nanoparticle	colorimetric	cortisol	2.8–7.3	2.5	[122]
lateral flow immunoassay	gold nanoparticles with silver enhancement	colorimetric	cortisol	0.5–150	0.5	[123]
lateral flow immunoassay	platinum nanoparticles	photoluminescence	cortisol	0.14–200	0.139	[124]
lateral flow immunoassay	HRP	chemiluminescence	cortisol	0.3–60	0.3	[125]
microchannel lateral flow assay on lab-on-a-chip	Dab-af488	fluorescence	cortisol	0.93–30.0	1.8	[126]
Immune- chromatographic test strip	fluorescent microsphere 300 nm	fluorescence	pepsin	2.5–100.0	1.9	[127]
multiple lateral flow imunostrip	green core–shell upconversion nanoparticles	luminescence	MMP-8, IL-1β and TNF-α	n/a	MMP-8: 5.455 IL-1β: 0.054 TNF-α: 4.439	[128]
lateral flow immunoplatform integrated with surface-enhanced Raman scattering	gold nanoparticle clusters with Raman reporter	SERS	cortisol	0.01 × 10^−3^−100	0.014 × 10^−3^	[129]
sandwich type of lateral flow immunoassay	enzymatic activity of streptavidin-alkaline phosphatase	electrochemical	CRP	n/a	3 (in buffer) 55 (in filtered saliva)	[130]
electrochemical lateral flow assay with nanocatalytic redox cycling	gold nanoparticles and ammonia-borane	electrochemical	insulin	0–29	0.07	[131]
vertical flow paper-based sandwich immunoassay	eosin-conjugated reporter molecules	colorimetric	MMP-8 and MMP-9	MMP-8: 0.02–0.85; MMP-9: 0.08–0.76	-	[132]

## Data Availability

No new data were created or analyzed in this study.
